# Marked improvement of isolated acrodermatitis continua of hallopeau with upadacitinib: a case report and literature review

**DOI:** 10.3389/fmed.2026.1868846

**Published:** 2026-07-24

**Authors:** Man Li, Gui-Xiang Zhang, Yong-Lan Wu, Hsin-Yi Huang

**Affiliations:** 1 Department of Hematology and Rheumatology, Longyan Second Hospital, Longyan, China; 2 Department of Dermatology, Kaohsiung Medical University Hospital, Kaohsiung Medical University, Kaohsiung, Taiwan

**Keywords:** acrodermatitis continua of Hallopeau, Janus kinase inhibitors, nail diseases, pustular psoriasis, upadacitinib

## Abstract

Acrodermatitis continua of Hallopeau (ACH) is a rare and chronic variant of pustular psoriasis characterized by sterile pustules, periungual inflammation, and progressive nail destruction. Due to its rarity and overlapping clinical features with other nail disorders, ACH is frequently underrecognized and remains difficult to treat, with no standardized therapeutic guidelines. Here, we report a case of long-standing, isolated ACH showing marked improvement after treatment with the selective Janus kinase 1 (JAK1) inhibitor upadacitinib. A 54-year-old man presented with a 13-year history of recurrent periungual erythema, sterile pustules, and progressive nail dystrophy affecting multiple digits. The disease was refractory to topical corticosteroids, systemic antibiotics, acitretin, and 1 year of secukinumab therapy. Following initiation of upadacitinib 15 mg daily, rapid clinical improvement was observed within 4 weeks, with reduction of subungual pustules and periungual inflammation. Nail involvement subsequently improved gradually, accompanied by nail regrowth and a decrease in the modified Nail Psoriasis Severity Index score from 66 at baseline to 16 at week 24 and 13 at month 9. No adverse or unanticipated events were observed during follow-up. This case highlights the potential role of JAK1 inhibition as an effective therapeutic strategy for refractory ACH and supports further investigation into JAK-targeted therapies in pustular psoriasis spectrum disorders.

## Introduction

Acrodermatitis continua of Hallopeau (ACH) is a rare variant of pustular psoriasis characterized by chronic sterile pustules affecting the distal digits and nail apparatus (1). Persistent inflammation may result in onychodystrophy, anonychia, and even osteolysis in severe cases (1). Due to its low prevalence and heterogeneous presentation, ACH is often misdiagnosed or delayed in diagnosis, leading to prolonged disease burden (2).

The pathogenesis of ACH is not fully elucidated but is increasingly recognized as involving dysregulated innate immune responses, particularly the interleukin (IL)-36 signaling pathway (2). Aberrant activation of IL-36 promotes recruitment of neutrophils and amplification of inflammatory cascades, contributing to pustule formation (2). While mutations in interleukin-36 receptor antagonist (IL36RN) and related genes have been identified in some patients, many cases remain genetically undefined, suggesting additional immune mechanisms (1, 2).

Therapeutic management of ACH remains challenging (1). Conventional treatments, including topical corticosteroids, retinoids, methotrexate, and cyclosporine, often yield inconsistent responses (1). Biologic agents targeting TNF-*α*, IL-17, and IL-23 pathways have demonstrated efficacy in selected cases; however, treatment resistance and relapse are common (1).

Janus kinase (JAK) signaling represents a shared downstream pathway of multiple cytokines implicated in ACH, including IL-6, interferons, and IL-36–related pathways (1). JAK inhibitors have shown efficacy in various inflammatory dermatoses, but evidence in ACH remains limited (3–7).

Herein, we present a case of refractory, isolated ACH that showed marked clinical improvement following treatment with upadacitinib and review the emerging role of JAK inhibition in this disease.

## Case description

A 54-year-old man presented with a 13-year history of recurrent periungual erythema, sterile pustules, and progressive nail dystrophy involving multiple distal digits of both hands. The disease initially developed at the distal phalanx of the left ring finger without an identifiable trigger and gradually extended bilaterally (Figures 1a,b). The modified Nail Psoriasis Severity Index (mNAPSI) score was 66, calculated across the six affected fingernails. The patient denied any history of plaque psoriasis, flexural psoriasis, psoriatic arthritis, or systemic comorbidities.

**Figure 1:**
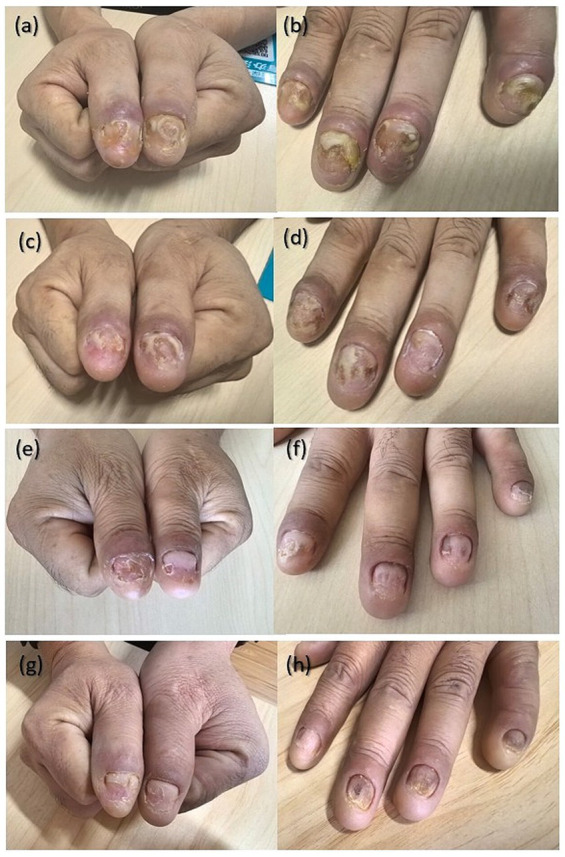
Clinical evolution of acrodermatitis continua of Hallopeau treated with Upadacitinib. **(a,b)** Baseline periungual erythema, subungual pustules, onycholysis, and nail dystrophy. **(c,d)** Week 4: Reduced pustulation and periungual inflammation. **(e,f)** Week 12: Marked improvement with gradual nail regrowth. **(g,h)** Week 24: Substantial improvement in pustulation and nail morphology, with mild residual nail dystrophy.

The patient initially received empiric oral moxifloxacin 400 mg once daily for 2 weeks, without significant improvement. Topical 0.05% halometasone cream was subsequently applied to the affected periungual lesions twice daily during flares, typically for two to 4 weeks, with only transient symptomatic relief and no sustained clinical improvement. Oral acitretin 25 mg once daily was then administered for 8 weeks, also with limited clinical response.

A periungual skin biopsy had been performed during the disease course, which demonstrated features consistent with acrodermatitis continua of Hallopeau, including regular acanthosis, confluent parakeratosis, hypogranulosis, and neutrophilic collections within the stratum corneum and upper epidermis (Figures 2a,b). Periodic acid–Schiff and Gram stains were negative for fungal and bacterial organisms (Figures 2c,d).

**Figure 2:**
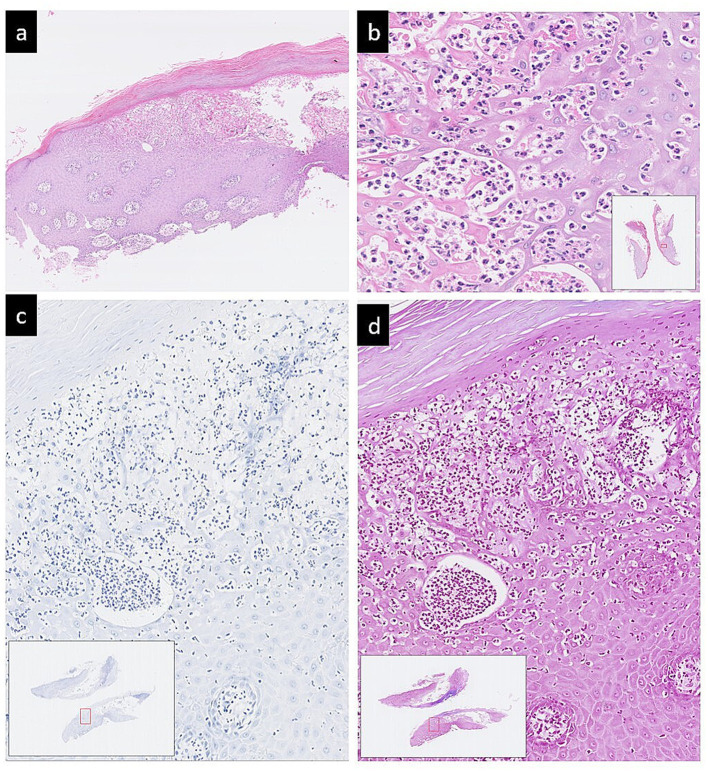
Histopathological findings of the periungual lesion **(a)** Hematoxylin and eosin staining showed psoriasiform epidermal hyperplasia with confluent parakeratosis and hypogranulosis (20x). **(b)** Higher-power view revealed prominent neutrophilic infiltration, with neutrophilic collections within the stratum corneum and upper epidermis (100x). **(c)** Periodic acid–Schiff staining was negative for fungal organisms (40x). **(d)** Gram staining was negative for bacterial organisms (40x).

Alternative diagnoses, including chronic paronychia, bacterial or fungal infection, nail psoriasis, eczema, and contact dermatitis, were carefully considered. The key differentiating features are summarized in Table 1. Chronic paronychia, eczema, and contact dermatitis were considered less likely because there was no definite history of chronic wet-work exposure, irritant or allergen contact, and the lesions showed only transient or poor response to topical corticosteroids and systemic antibiotics. Bacterial and fungal infections were excluded by unremarkable microbiological studies, including bacterial culture, fungal culture, and potassium hydroxide examination. In addition, PAS and Gram stains of the biopsy specimen were negative for fungal organisms and bacteria. Although nail psoriasis was considered, the patient had no concomitant plaque psoriasis or psoriatic arthritis, and the clinical presentation of recurrent sterile periungual pustules with progressive nail dystrophy, together with compatible histopathologic findings, supported the diagnosis of acrodermatitis continua of Hallopeau. Plain radiography of the affected hand showed no obvious osteolysis, distal phalangeal tuft resorption, erosive change, destructive arthropathy, or distal interphalangeal joint space narrowing.

**Table 1:** Differential diagnoses and key distinguishing features of acrodermatitis continua of Hallopeau in this case.

Differential diagnosis	Key distinguishing features	Reasons considered less likely in this case
Chronic paronychia	Chronic periungual inflammation, often associated with wet work, irritant exposure, or Candida colonization	No definite wet-work or irritant exposure; poor response to antibiotics and topical corticosteroids; fungal studies were negative
Bacterial or fungal infection	Positive culture, KOH, PAS, or Gram stain; may show suppurative infection	Bacterial culture, fungal culture, and KOH examination were unremarkable; PAS and Gram stains were negative
Nail psoriasis	Nail pitting, onycholysis, subungual hyperkeratosis; often associated with plaque psoriasis or psoriatic arthritis	No plaque psoriasis or psoriatic arthritis; recurrent sterile periungual pustules and compatible biopsy favored ACH
Eczema/contact dermatitis	History of irritant or allergen exposure; eczematous periungual dermatitis; usually improves with avoidance and topical therapy	No clear exposure history; only transient or poor response to topical corticosteroids
Parakeratosis pustulosa	Usually affects children; localized eczematous or psoriasiform disorder of one or few digits; limited course	Adult onset, multiple nail-unit involvement, 13-year progressive course, recurrent sterile pustules, and marked nail dystrophy
Chronic mucocutaneous candidiasis/immunodeficiency-associated nail disease	Persistent mucocutaneous candidiasis, recurrent fungal or opportunistic infections, or abnormal immune evaluation	No oral, genital, or other mucocutaneous candidiasis; no recurrent severe or opportunistic infections; HIV screening and baseline laboratory tests were unremarkable

The patient was subsequently treated with secukinumab (300 mg) for 1 year; however, the disease remained relapsing with persistent pustulation and progressive nail destruction. Given the refractory course, treatment was switched to oral upadacitinib 15 mg once daily. Baseline safety evaluation was performed, and laboratory monitoring, including complete blood count, renal and liver function tests, lipid profile, and infection screening, remained within normal limits throughout treatment.

Clinical improvement was observed as early as week 4, with reduction of subungual pustules and periungual inflammation, and mNAPSI score decreased to 54 at this time point (Figures 1c,d). A marked improvement in nail involvement was noted by week 12, with the mNAPSI score decreasing to 25, consistent with gradual nail regrowth (Figures 1e,f). By week 24, pustulation had largely resolved and nail morphology had substantially improved, with further reduction of the mNAPSI score to 16 (Figures 1g,h). After 9 months of continuous therapy, clinical improvement was maintained, with a residual mNAPSI score of 13 and no adverse or unanticipated events. The longitudinal changes in mNAPSI and pain VAS scores during secukinumab and subsequent upadacitinib treatment are illustrated in Figure 3.

**Figure 3:**
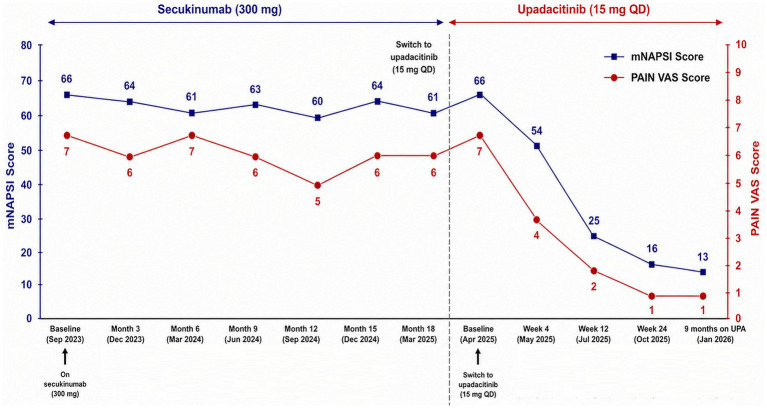
Longitudinal changes in Modified Nail Psoriasis Severity Index (mNAPSI) and Pain Visual Analog Scale (VAS) scores during treatment. During Secukinumab therapy, disease activity remained persistently elevated with fluctuating scores. Following the switch to Upadacitinib (15 mg once daily), both mNAPSI and Pain VAS scores showed rapid and sustained improvement over time.

From the patient’s perspective, the long-standing disease had a substantial impact on daily life. Before upadacitinib treatment, multiple nail units were severely damaged, and recurrent pustulation limited daily activities such as writing, household tasks, and grasping objects. Because of pain and the visible appearance of the lesions, the patient also avoided social interactions. After 4 weeks of upadacitinib treatment, he was able to independently perform personal hygiene activities and light manual work. By week 24, the appearance and function of his hands had largely recovered, and his social activities and work were no longer substantially limited. The patient reported a marked improvement in quality of life and, compared with multiple previously ineffective treatments, was highly satisfied with the rapid and sustained response to upadacitinib.

## Discussion

ACH is a rare pustular disorder with limited evidence guiding treatment decisions (1, 2). Due to its chronic relapsing course and destructive impact on the nail unit, and overlap with other inflammatory and infectious nail disorders, ACH may be difficult to recognize in clinical practice.

This case highlights several diagnostic challenges beyond the rarity of ACH. The isolated nail-unit and periungual presentation, absence of concomitant plaque psoriasis or psoriatic arthritis, and long relapsing disease course made early recognition difficult. In addition, the clinical overlap with chronic paronychia, bacterial or fungal infection, nail psoriasis, eczema/contact dermatitis, and parakeratosis pustulosa may further delay diagnosis. Therefore, clinicopathological correlation, exclusion of infection, radiographic assessment for bone or joint involvement, and careful longitudinal observation were important in establishing the diagnosis.

Achieving durable disease control also remains a major clinical challenge. Conventional systemic therapies, including retinoids, methotrexate, and cyclosporine, show variable efficacy. Biologic therapies targeting TNF-*α* and IL-17 pathways have expanded treatment options, yet a significant proportion of patients exhibit incomplete responses or relapse after initial improvement (7–9).

Emerging evidence suggests that the IL-36 pathway plays a central role in pustular psoriasis spectrum disorders, including ACH (10–12). Targeting this pathway with spesolimab has demonstrated promising results, including rapid resolution of pustules regardless of IL36RN mutation status, as reported in recent case studies (13). However, access, cost, and long-term data remain limitations. IL36RN genetic testing was unavailable at our institution, representing a limitation of this report. Although spesolimab is reimbursed in China for adult GPP flares, it is not reimbursed for isolated chronic ACH and was therefore not selected because of cost and feasibility concerns.

The rationale for using a JAK1 inhibitor in this patient was based on both clinical and mechanistic considerations. Clinically, the patient had long-standing isolated ACH that was refractory to topical corticosteroids, systemic antibiotics, acitretin, and 1 year of IL-17A inhibition with secukinumab. Given the persistent relapsing pustulation and progressive nail destruction, an alternative systemic therapy with broader immunomodulatory effects was considered.

JAK signaling integrates multiple cytokine pathways involved in ACH pathogenesis (14). Upadacitinib, a selective JAK1 inhibitor, may exert therapeutic effects in ACH by targeting upstream cytokine signaling pathways that converge on the JAK–STAT axis (14). In ACH, disease pathogenesis involves a complex cytokine network comprising IL-36-driven innate immune activation together with downstream amplification by IL-6, TNF-*α*, IL-23/IL-17, and interferon pathways (1, 2). By selectively inhibiting JAK1, upadacitinib can simultaneously attenuate multiple proinflammatory signals, particularly IL-6 pathways, thereby reducing neutrophil recruitment, keratinocyte activation, and inflammatory amplification (14). Although cytokine profiling, phosphorylated STAT assays, and lesional immunohistochemistry were not performed in our patient, previous basic laboratory studies have shown that upadacitinib inhibits JAK1-dependent cytokine signaling, including IL-6-induced STAT3 phosphorylation, supporting the biologic plausibility of this proposed mechanism (15).

Compared with single-cytokine-targeted biologics, upadacitinib provides broader immunomodulation at an upstream signaling level, which may explain its efficacy in refractory ACH or cases with incomplete response to conventional biologics. This multi-pathway suppression is particularly relevant in ACH, where IL-36-mediated inflammation triggers a cascade of downstream cytokine responses rather than a single dominant pathway.

To date, only a limited number of cases have reported the use of JAK or TYK2 inhibitors in ACH (Table 2) (3–7). Our case demonstrates marked improvement of long-standing, isolated ACH after initiation of upadacitinib, following an incomplete and fluctuating response to IL-17A inhibition. However, this observation should be interpreted cautiously. As a single case report, it does not allow conclusions regarding the relative efficacy of JAK1 inhibition compared with IL-17A inhibition. Rather, the response observed in this patient represents an individual clinical observation and adds to the limited clinical experience with JAK inhibition in refractory ACH. Further studies are required to clarify the therapeutic role, efficacy, and optimal positioning of JAK1 inhibition in ACH treatment.

**Table 2:** Reported cases of acrodermatitis continua of Hallopeau treated with Janus kinase or TYK2 inhibitors.

Author, (year)	Age/Sex	ACH characteristics	Concomitant psoriasis/arthritis	ACH duration	JAK/TYK2 inhibitor (dose)	Follow up duration	Previous therapies	Time to response	Outcome
Han et al. (3)	28 / F	Fingernail pustules, DIP joint swelling, bone erosions	Arthritic involvement	Not reported	Baricitinib 2 mg/day	5 months	Antibiotics, acitretin, methotrexate	5 days	Rapid clinical improvement; sustained remission for 5 months with no progression of bone erosions
Kan and Uhara (4)	74 / M	Chronic ACH affecting fingers and toes with severe nail dystrophy	None	≥ 2 years	Deucravacitinib 6 mg every other day	24 weeks	Acitretin, cyclosporine, tonsillectomy	< 8 weeks	Marked improvement of skin and nail lesions; near-complete remission by 24 weeks
Muto et al. (6)	70 / M	Painful pustules, nail deformities, erosions	None	Not reported	Baricitinib 4 mg/day	4 weeks	Topical steroids, vitamin D, antibiotics, apremilast, etretinate, deucravacitinib	4 weeks	Significant reduction of lesions; no recurrence or adverse events
Shen et al. (5)	47 / F	Periungual pustules with nail loss and dystrophy	None	1 year	Tofacitinib 5 mg twice daily	12 weeks	Topical calcipotriol/betamethasone	4 weeks	Progressive nail regrowth and skin clearance; near-complete resolution at 12 weeks
Yang et al. (7)	52 / M	Long-standing, refractory ACH with nail destruction	Flexural psoriasis, arthritis	10 years	Upadacitinib 15 mg/day (later q3d)	9 months	Acitretin, apremilast, ixekizumab, adalimumab, secukinumab, guselkumab	1–2 weeks	Rapid and complete remission of ACH and joint symptoms; sustained control for 9 months
Present case	54/M	Chronic ACH of fingers with severe nail dystrophy	None	13 years	Upadacitinib 15 mg/day	9 months	Acitretin, topical corticosteroids, secukinumab	4 weeks	Marked improvement by 12 weeks, with complete pustule clearance and firm nail regrowth at 24 weeks

This report has several limitations. First, it describes a single patient, and the findings cannot be generalized. Second, genetic testing for pustular psoriasis-related genes, including IL36RN, was not performed because it was unavailable at our institution. Third, cytokine profiling, phosphorylated STAT assays, and lesional JAK–STAT pathway analysis were not conducted, limiting mechanistic interpretation. Finally, although clinical improvement was maintained for 9 months, longer follow-up and additional cases are needed to determine the durability, safety, and reproducibility of upadacitinib treatment in isolated ACH.

## Conclusion

In this case, long-standing, biologic-refractory acrodermatitis continua of Hallopeau showed rapid and sustained clinical remission following treatment with upadacitinib. This finding highlights the potential role of JAK1 inhibition as a multi-pathway therapeutic approach in ACH, particularly in patients with inadequate response to conventional biologic therapies. Further studies are warranted to evaluate its long-term efficacy and safety in ACH.

## Data Availability

The raw data supporting the conclusions of this article will be made available by the authors, without undue reservation.
